# Decrease of Plasma Glucose by *Hibiscus taiwanensis* in Type-1-Like Diabetic Rats

**DOI:** 10.1155/2013/356705

**Published:** 2013-04-17

**Authors:** Lin-Yu Wang, Hsien-Hui Chung, Juei-Tang Cheng

**Affiliations:** ^1^Department of Pediatrics, Chi-Mei Medical Center, Yong Kang, Tainan City 73101, Taiwan; ^2^The Center of General Education, Chia Nan University of Pharmacy & Science, Rende, Tainan City 71710, Taiwan; ^3^Institute of Basic Medical Sciences, College of Medicine, National Cheng Kung University, Tainan City 70101, Taiwan; ^4^Graduate Institute of Medical Sciences, Chang Jung Christian University, Quei-Jen, Tainan City 71101, Taiwan; ^5^Department of Medical Research, Chi-Mei Medical Center, Yong Kang, Tainan City 73101, Taiwan

## Abstract

*Hibiscus taiwanensis* (Malvaceae) is widely used as an alternative herb to treat disorders in Taiwan. In the present study, it is used to screen the effect on diabetic hyperglycemia in streptozotocin-induced diabetic rats (STZ-diabetic rats). The extract of *Hibiscus taiwanensis* showed a significant plasma glucose-lowering action in STZ-diabetic rats. Stems of *Hibiscus taiwanensis* are more effective than other parts to decrease the plasma glucose in a dose-dependent manner. Oral administration of *Hibiscus taiwanensis* three times daily for 3 days into STZ-diabetic rats increased the sensitivity to exogenous insulin showing an increase in insulin sensitivity. Moreover, similar repeated administration of *Hibiscus taiwanensis* for 3 days in STZ-diabetic rats produced a marked reduction of phosphoenolpyruvate carboxykinase (PEPCK) expression in liver and an increased expression of glucose transporter subtype 4 (GLUT 4) in skeletal muscle. Taken together, our results suggest that *Hibiscus taiwanensis* has the ability to lower plasma glucose through an increase in glucose utilization via elevation of skeletal GLUT 4 and decrease of hepatic PEPCK in STZ-diabetic rats.

## 1. Introduction

Diabetes is a well-known metabolic disease and often leads to many physiological complications, including cardiovascular diseases, renal diseases, and retinal damage [1–3]. Recently, the management of diabetic hyperglycemia has attracted much attention in alternative aspect [4, 5]. Some agents from herbal plants are thought to improve diabetic hyperglycemia [6–8]. Thus, alternative medicine and other herbal supplements for handling of diabetic disorders are necessary.

*Hibiscus taiwanensis *S. Y. Hu (Malvaceae) is native to Taiwan and widely distributed throughout the island. Recently, the stems and roots of *Hibiscus taiwanensis* have been used as anti-inflammatory, antifungal, antipyretic, and anthelmintic agents in traditional Chinese medicine [9, 10]. *Hibiscus taiwanensis* had anti-inflammatory action in vitro and in vivo via increasing the activities of catalase (CAT), superoxide dismutase (SOD), and glutathione peroxidase (GPx) [11]. Many active principles have been isolated from the stems of *Hibiscus taiwanensis*, and some of them showed cytotoxic activity against human carcinoma, including breast and lung cancer cells [12, 13]. Antioxidant-like substances are known to produce antidiabetic action [14, 15]. In recent, an active principle isolated from *Hibiscus taiwanensis* named syringaldehyde showed a plasma glucose-lowering action in streptozotocin-induced diabetic rats (STZ-diabetic rats) [16]. However, the effect of *Hibiscus taiwanensis* on diabetic disorders remained obscure.

In the present study, we thus employed STZ-diabetic rats as an animal model of type-1-like diabetic disorders to screen the changes in plasma glucose and clarify the potential mechanism for this action. The main aim is going to provide a new insight of alternative medicine into the improvement of diabetic hyperglycemia.

## 2. Materials and Methods

### 2.1. Plant Materials

The extract with 60% aqueous acetone of *Hibiscus taiwanensis* was provided by Hercet Co. Ltd. (Kaohsiung, Taiwan). The plant material was identified by Professor M. I. Wu (Kaohsiung Committee of Chinese Medicine; Kaohsiung City, Taiwan). A voucher specimen (BT-H-00151) was deposited in the herbarium of the Agricultural Research Institute (Taichung, Taiwan). 

### 2.2. Preparation of Plant Extracts

In the present study, the plant parts were used to compare their plasma glucose-lowering action, including leaf, stem, and fruit. Dried each part of *Hibiscus taiwanensis* (1.5 kg) was extracted with 7 L of aqueous acetone solution (60%) by maceration at room temperature for three days. The extraction process was repeated three times. The extract was concentrated under reduced pressure at 40°C, yielding 1.5 L of an aqueous extract, and it was diluted to the desired concentration when used.

### 2.3. Animal Models

Ten-week-old male Wistar rats weighing 250 to 300 g were obtained from the Animal Center of National Cheng Kung University Medical College. The diet of the animals used for the study was standard laboratory diet. The number of animals for each group of experiment is eight. STZ-diabetic rats were induced by intravenous injection (IV) of STZ (65 mg/kg) into Wistar rats according to the previous method [17]. Animals were considered to be diabetic if they had plasma glucose concentrations of 320 mg/dL or greater in addition to polyuria and other diabetic features. All studies were carried out 2 weeks after the injection of STZ. All animal procedures were performed according to the Guide for the Care and Use of Laboratory Animals of the National Institutes of Health, as well as the guidelines of the Animal Welfare Act. In our preliminary data, the effective dose of the plant extract in reducing the plasma glucose in type-1 diabetic rats was 500 mg/kg, and the volume of the extract was administered according to the body weight of the rat (mL/kg). Also, the treated dose of each part was 500 mg/kg.

### 2.4. Laboratory Determinations

The determination of plasma glucose was conducted according to the previous study [18]. The concentration of plasma glucose was measured by the glucose oxidase method using an analyzer (Quik-Lab, Ames; Miles Inc., Elkhart, IN, USA). 

### 2.5. Measurement of Insulin Sensitivity in Rats

Because the STZ-induced diabetic rats used in the present study were negligible for endogenous insulin, the plasma glucose-lowering action depended on the action of exogenous insulin in STZ-induced diabetic rats. The obtained results can thus be used to indicate the insulin sensitivity. These rats received an injection of long-acting human insulin at 1 IU/kg once daily to normalize the insulin sensitivity. Then, three days later, the STZ-diabetic rats were divided into two groups. One group received the oral treatment of *Hibiscus taiwanensis* at 500 mg/kg dissolving in saline solution, three times daily (t.i.d.), and another group received similar treatment with the same volume of saline. After three days of treatment, all rats were used to challenge with exogenous insulin. According to a previous method [16], an intravenous insulin challenge test was performed by giving 0.1 to 1.0 IU/kg of short-acting human insulin into these STZ-diabetic rats. Blood samples (0.2 mL) from the femoral vein were drawn at 30 min following the intravenous insulin challenge test for the measurement of plasma glucose concentrations.

### 2.6. Quantitative Reverse Transcription-Polymerase Chain Reaction (qRT-PCR)

Total RNA was extracted from liver and soleus muscle tissue samples using Trizol reagent (Invitrogen). Two microgram of total RNA was used for the reverse transcription reaction, along with Superscriptase II (Invitrogen), oligo-dT, and random primers. The web-based assay design software from the Universal Probe Library Assay Design Center was used to design the TaqMan primer pairs and to select the appropriate hybridization probes. The reactions were performed in 20 *μ*L of a mixture consisting of 13.4 *μ*L of PCR buffer, 0.2 *μ*L of each probe (20 *μ*mol/L), 4 *μ*L of LightCycler TaqMan (Roche Diagnostics GmbH), and 2 *μ*L of template cDNA. A LightCycler Detection System (Roche Applied Science) was used for amplification and detection. The PCR reaction was carried out as follows: one cycle of 95°C for 10 min, 45 cycles of 94°C for 10 s, 60°C for 20 s, and 72°C for 1 s. The crossing point for each amplification curve was determined using the second derivative maximum method. The concentration of each gene was calculated with the aid of the LightCycler software using the respective standard curve as reference. Relative gene expression was expressed as a ratio of the target gene concentration to the housekeeping gene 36B4 concentration. 

### 2.7. Western Blotting Analysis

Western blotting analysis was carried out as previously described [19] and quantification was obtained from three individual experiments. After homogenization of liver and soleus muscle using a glass/Teflon homogenizer, the homogenates (50 *μ*g) were separated by sodium dodecyl sulfate-polyacrylamide gel electrophoresis, and western blot analysis was performed using either an anti-rat GLUT 4 antibody purchased from (Abcam, Cambridge, UK) in soleus muscle or an anti-rat PEPCK antibody from Santa Cruz Biotechnology, CA, USA, in liver. The blots were probed with a goat polyclonal actin antibody from (Millipore, Billerica, MA, USA) to ensure that the amount of protein loaded into each lane of the gel was constant. Blots were incubated with the appropriate peroxidase-conjugated secondary antibodies. After removal of the secondary antibodies, the blots were washed and developed using the ECL-Western blotting system. Densities of the obtained immunoblots at 45 KDa for GLUT 4, 69.5 KDa for PEPCK, and 43 KDa for actin were quantified using laser densitometer.

### 2.8. Statistical Analysis

The plasma glucose-lowering activity of *Hibiscus taiwanensis* was calculated as the percentage decrease of the initial glucose value according to the following formula: (*G*_*i*_ − *G*_*t*_)/*G*_*i*_ × 100%, where *G*_*i*_ is the initial glucose concentration and *G*_*t*_ is the plasma glucose concentration after treatment of* Hibiscus taiwanensis*. Data are expressed as the mean ± S.E.M. for the number (*n*) of animals in the group as indicated in tables and figures. Differences among groups were analyzed by one-way ANOVA. The Dunnett range post hoc comparisons were used to determine the source of significant differences where appropriate. A *P* value of 0.05 or less was considered statistically significant.

## 3. Results

### 3.1. Comparison of Effects of Various Parts Prepared from *Hibiscus taiwanensis* on Plasma Glucose Concentration in STZ-Diabetic Rats

**Figure 1:**
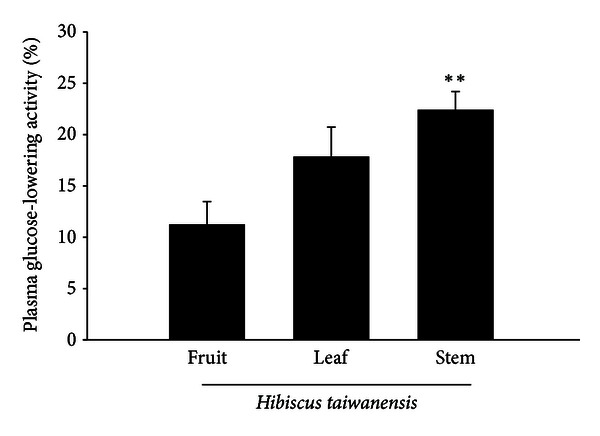
Comparison of the glucose-lowering activity between the extracts from fruit, leaf, or stem of *Hibiscus taiwanensis*. Values of mean and bar of S.E.M. were obtained from each group of eight rats. Vehicle only (0.9% saline) was given at the same volume. **P < 0.01 versus data from animals treated with vehicle (0).

As shown in Figure 1, the plasma glucose-lowering activities were 11.2 ± 2.3%, 17.8 ± 2.9%, and 22.4 ± 1.8% in STZ-diabetic rats receiving oral intake of extract (500 mg/kg) prepared from fruit, leaf, and stem of *Hibiscus taiwanensis*, respectively (*n* = 8). The stems show a better plasma-lowering action than other parts. Thus, the following experiments were performed using the extract of stems from *Hibiscus taiwanensis*.

### 3.2. Dose-Dependent Action of *Hibiscus taiwanensis* to Lower Plasma Glucose in STZ-Diabetic Rats

**Figure 2:**
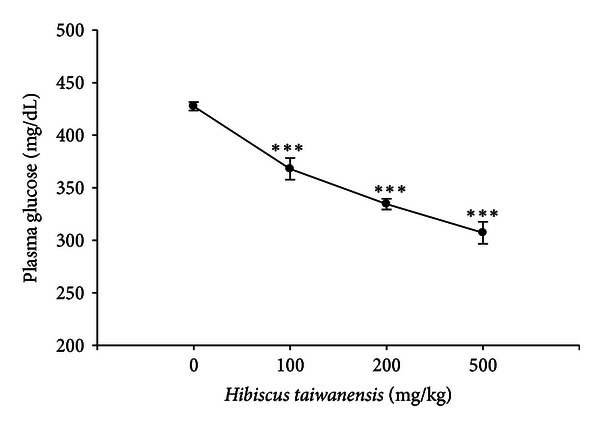
The plasma glucose-lowering activity produced by an oral intake of the stem extract from *Hibiscus taiwanensis* in STZ-diabetic rats. Values of mean and bar of S.E.M. were obtained from each group of eight rats. Vehicle only (0.9% saline) was given at the same volume. ***P < 0.001 versus data from animals treated with vehicle (0).

Ninety minutes after treatment, the plasma glucose-lowering activities were 15.1 ± 2.0%, 22.1 ± 2.4%, and 28.9 ± 1.9% in STZ-diabetic rats receiving oral intake of *Hibiscus taiwanensis* at 100 mg/kg, 200 mg/kg and 500 mg/kg, respectively (*n* = 8). As shown in Figure 2, a dose-dependent reduction of plasma glucose by *Hibiscus taiwanensis* was observed and it significantly decreased the plasma glucose concentration to 307.2 ± 10.5 mg/dL (*P* < 0.001; *n* = 8) at 500 mg/kg. As the positive control, treatment with metformin in various doses (50 mg/kg, 75 mg/kg, and 100 mg/kg) attenuated the plasma glucose from 431.8 ± 5.6 mg/dL to 356.6 ± 3.4 mg/dL, 326.6 ± 2.9 mg/dL, and 294.9 ± 3.7 mg/dL (*n* = 8) showing 17.3 ± 1.3%, 24.2 ± 1.4%, and 31.6 ± 1.3% plasma glucose-lowering activities, respectively.

### 3.3. Effect of *Hibiscus taiwanensis* on Insulin Sensitivity in STZ-Induced Diabetic Rats

**Figure 3:**
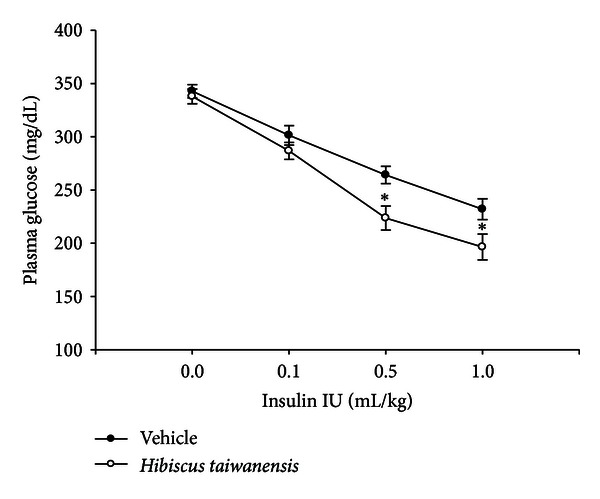
Effect of *Hibiscus taiwanensis* on insulin sensitivity in STZ-diabetic rats. *Hibiscus taiwanensis* at 500 mg/kg was treated orally into STZ-diabetic rats three times daily for three days. Then, the animals were injected intravenously with exogenous insulin at the indicated dose to show the changes in plasma glucose as open circles. Changes of plasma glucose in another group of STZ-diabetic rats receiving a similar treatment with vehicle at the same volume are shown as closed circles. Values (means ± SE) were obtained from each group of eight animals. *P < 0.05 as compared with values from vehicle-treated group (closed circles) at the same dose of insulin.

The change of insulin sensitivity was investigated in STZ-induced diabetic rats. The basal plasma glucose concentration in STZ-diabetic rats was 334.9 ± 7.2 mg/dL. The plasma glucose-lowering activity of short-acting human insulin (exogenous insulin) at doses from 0.1 to 1.0 IU/kg in STZ-diabetic rats receiving oral intake of *Hibiscus taiwanensis* (500 mg/kg, three times daily) for 3 days was markedly higher than that in the control group receiving same volume of vehicle (Figure 3). The plasma glucose-lowering activity of exogenous insulin in the *Hibiscus taiwanensis*-treated group (500 mg/kg) was about 34.0 ± 2.8% at 0.5 IU/kg and more markedly 42.0 ± 3.1% at the dose of 1.0 IU/kg (Figure 3). An increase in insulin sensitivity by *Hibiscus taiwanensis* can be identified.

### 3.4. Effect of *Hibiscus taiwanensis* on Changes of GLUT 4 in Skeletal Muscle of STZ-Diabetic Rats

**Figure 4:**
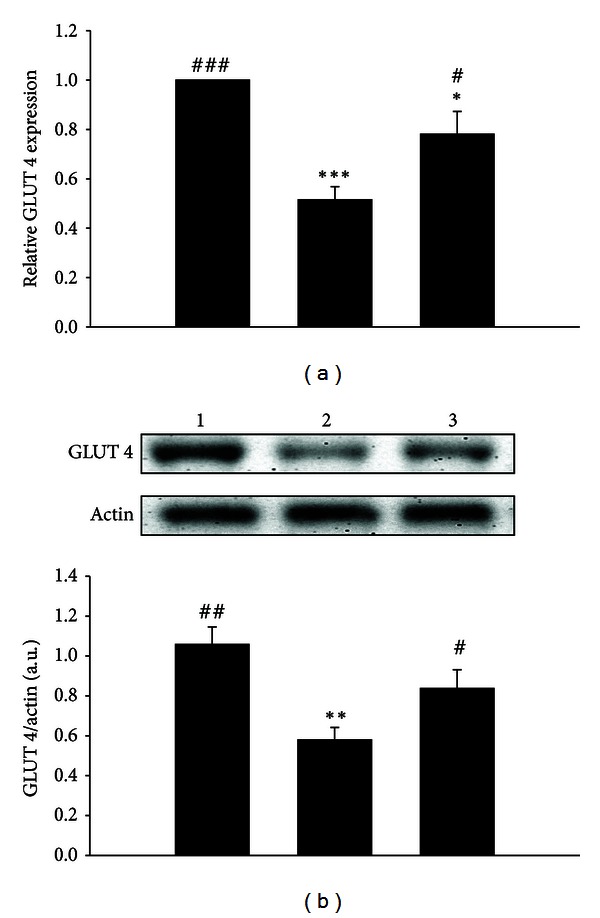
The relative GLUT 4 expression in skeletal muscle isolated from STZ-diabetic rats received oral intake with *Hibiscus taiwanensis* (500 mg/kg) three times daily for 3 days. (a) Lane 1, vehicle-treated Wistar rats; lane 2, vehicle-treated STZ-diabetic rats; lane 3, *Hibiscus taiwanensis* (500 mg/kg)-treated STZ-diabetic rats. The samples were then collected for qRT-PCR. Data expressed as mean with standard error (SE) (*n* = 6 per group) is indicated in each column. *P < 0.05 and ***P < 0.001 compared with data obtained from lane 1. ^#^ *P* < 0.05 and ^###^ *P* < 0.001 compared with data obtained from lane 2. (b) Upper panel shows the representative response of protein level for GLUT 4 or actin in skeletal muscle isolated from STZ-diabetic rats receiving treatment with *Hibiscus taiwanensis* three times daily for 3 days. Lane 1, vehicle-treated Wistar rats; lane 2, vehicle-treated STZ-diabetic rats; lane 3, *Hibiscus taiwanensis* (500 mg/kg)-treated STZ-diabetic rats. Quantification of protein level using GLUT 4/actin expressed as mean with standard error (SE) (*n* = 6 per group) in each column is indicated in the lower panel. **P < 0.01 compared with data obtained from lane 1. ^#^ *P* < 0.05 and ^##^ *P* < 0.01 compared with data obtained from lane 2.

Treatment of STZ-diabetic rats with oral intake of *Hibiscus taiwanensis* (500 mg/kg) three times daily for 3 days resulted in an elevation of GLUT 4 mRNA level in skeletal muscle (Figure 4(a)). Western blot analysis showed a similar effect of *Hibiscus taiwanensis* (500 mg/kg) on the changes of GLUT 4 protein level in skeletal muscle (Figure 4(b)).

### 3.5. Effect of *Hibiscus taiwanensis* on Changes of Hepatic PEPCK in STZ-Diabetic Rats

**Figure 5:**
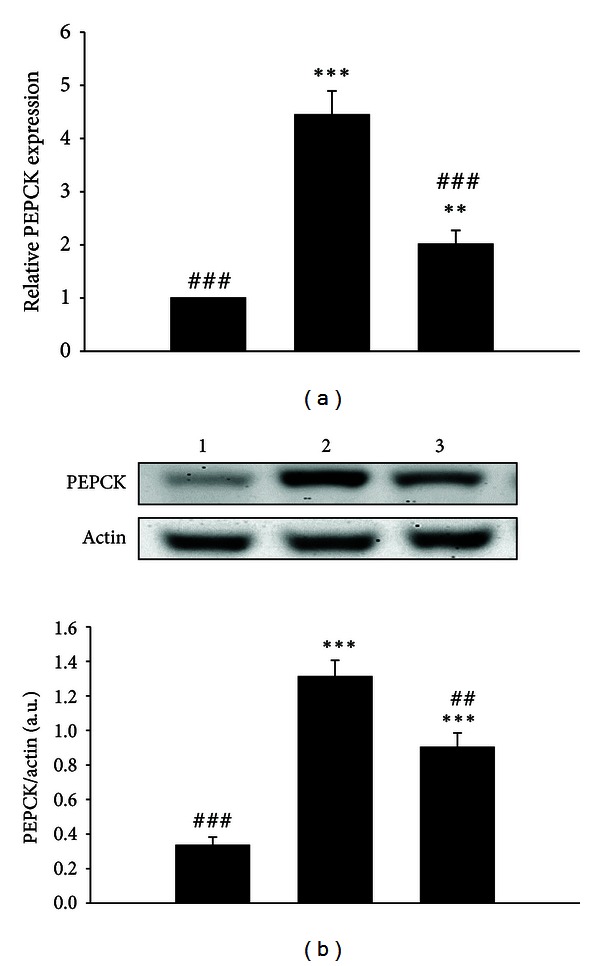
The relative PEPCK expression in liver isolated from STZ-diabetic rats received oral intake with *Hibiscus taiwanensis* (500 mg/kg) three times daily for 3 days. (a) Lane 1, vehicle-treated Wistar rats; lane 2, vehicle-treated STZ-diabetic rats; lane 3, *Hibiscus taiwanensis* (500 mg/kg)-treated STZ-diabetic rats. The samples were then collected for qRT-PCR. Data expressed as mean with standard error (SE) (*n* = 6 per group) is indicated in each column. **P < 0.01 and ***P < 0.001 compared with data obtained from lane 1. ^###^ *P* < 0.001 compared with data obtained from lane 2. (b) Upper panel shows the representative response of protein level for PEPCK or actin in liver isolated from STZ-diabetic rats receiving treatment with *Hibiscus taiwanensis* three times daily for 3 days. Lane 1, vehicle-treated Wistar rats; lane 2, vehicle-treated STZ-diabetic rats; lane 3, *Hibiscus taiwanensis* (500 mg/kg)-treated STZ-diabetic rats. Quantification of protein level using PEPCK/actin expressed as mean with standard error (SE) (*n* = 6 per group) in each column is indicated in the lower panel. ***P < 0.001 compared with data obtained from lane 1. ^##^ *P* < 0.01 and ^###^ *P* < 0.001 compared with data obtained from lane 2.

In the present study, the mRNA level of PEPCK in liver of STZ-diabetic rats was raised to about 4.4-folds of that in nondiabetic rats. The reduction of PEPCK mRNA level by *Hibiscus taiwanensis* in diabetic rats was observed (Figure 5(a)). Similarly, the protein level of PEPCK in liver of STZ-diabetic rats was raised to approximately 4.0-folds of that in nondiabetic rats. The protein level of PEPCK in diabetic rats was also reversed by *Hibiscus taiwanensis* to normal level (Figure 5(b)).

## 4. Discussion

In the present study, we found that the extract of herb named *Hibiscus taiwanensis* at 500 mg/kg has an ability to improve diabetic hyperglycemia in animal. Also, the stems show more effective plasma glucose-lowering action than fruit and leaf. This is consistent with previous report showing actions of stems [12, 13] in a dose-dependent manner. In addition, *Hibiscus taiwanensis *increased the insulin sensitivity to improve diabetic hyperglycemia via the regulation of peripheral glucose utilization and hepatic glucose output in STZ-diabetic rats.

In the present study, we employed STZ-diabetic rats to screen the effectiveness of herbal extract. This model belongs to type-1-like diabetic disorders due to insulin deficiency as described previously [20]. Although we did not check the plasma insulin level, the higher plasma glucose supports the success of this model similar to our reports [21, 22]. Diabetic hyperglycemia is resulted mainly from the dysfunction in glucose homeostasis while insulin sensitivity plays a critical role in the regulation of diabetic hyperglycemia [23]. Some agents useful in the improvement of diabetic hyperglycemia are believed to increase insulin sensitivity [24–26]. According to the previous method [16], *Hibiscus taiwanensis* was orally administered into diabetic rats at 500 mg/kg three times per day for three days. Then, the responses to exogenous insulin were compared with the vehicle-treated group. As shown in Figure 3, responses to exogenous insulin were markedly increased by treatment with *Hibiscus taiwanensis* in STZ-diabetic rats. It means that *Hibiscus taiwanensis* elevated the sensitivity of peripheral tissues to insulin directly because type-1-like diabetic rats lack endogenous insulin. An increase of insulin sensitivity by *Hibiscus taiwanensis* can thus be considered. This is useful to apply in insulin resistance and/or type-2 diabetic disorders widely observed in clinics [27]. However, the molecular mechanism for *Hibiscus taiwanensis* to increase insulin sensitivity shall be investigated in the future. 

In addition to insulin deficiency, diabetic hyperglycemia is thought to be the consequence of increased hepatic glucose output and reduced peripheral glucose utilization [28, 29]. Many related metabolic proteins were involved in the diabetic hyperglycemia, including AMP Kinase (AMPK), GLUT 4, PEPCK, acetyl CoA carboxylase, glycogen synthase kinase-3, and glycogen synthase [30]. Among them, GLUT 4 and PEPCK were commonly used to investigate diabetic disorders [31], and plasma glucose-lowering activities of some agents were associated with an increase in the glucose utilization in peripheral tissues and a reduction in hepatic gluconeogenesis [19, 32]. Glucose transport, which depends on insulin-stimulated translocation of glucose carriers to the cell membrane, is the rate-limiting step in the glucose metabolism of skeletal muscle [33]. The decreased expression of skeletal muscle GLUT 4 was previously proposed to cause the reduction of insulin-mediated glucose utilization in diabetic skeletal muscle [34–36]. Hepatic gluconeogenesis has an important influence on glucose metabolism [37]. Additionally, insulin deficiency is closely correlated with hepatic glucose output via increased expression of PEPCK that is a key enzyme of hepatic carbohydrate metabolism in diabetic hyperglycemia [28, 38, 39]. Liver-specific inhibition of PEPCK with RNAi improved diabetic hyperglycemia [40]. It is worthwhile to investigate whether *Hibiscus taiwanensis* exerted its antihyperglycemic action in diabetic rats by overturning the diabetes-dependent reduction of GLUT 4 expression and increased PEPCK expression. To provide ample time for alterations in gene expression, STZ-diabetic rats received repeated *Hibiscus taiwanensis* treatments for 3 days. As shown in Figures 4(a) and 5(a), the mRNA level of GLUT 4 was raised and the mRNA level of PEPCK was reduced by this herbal extract at dose sufficient to produce plasma glucose-lowering action; this change is similar to the action of effective agent showed in previous studies [41, 42]. Moreover, the lowered GLUT 4 protein level due to diabetes was also elevated by treatment with *Hibiscus taiwanensis* (Figure 4(b)). Similarly, the increased hepatic PEPCK protein by diabetes was attenuated by *Hibiscus taiwanensis *(Figure 5(b)). Thus, under an insulin-independent condition, *Hibiscus taiwanensis *modulated the gene expressions of muscle GLUT 4 and hepatic PEPCK of both mRNA and protein levels.

## 5. Conclusions

In conclusion, our results suggest that *Hibiscus taiwanensis* is merit for diabetic hyperglycemia mainly mediated by the enhancement of GLUT 4 gene expression and/or the amelioration of hepatic PEPCK gene expression. Therefore, *Hibiscus taiwanensis* can be used as the alternative agent for improvement of diabetic hyperglycemia. 

## Abbreviations

GLUT 4: Glucose transporter subtype 4
PEPCK: Phosphoenolpyruvate carboxykinase
STZ: Streptozotocin.
